# The Inverse Poisson Functional for forecasting response time to environmental events and global climate change

**DOI:** 10.1038/s41598-018-29680-4

**Published:** 2018-07-27

**Authors:** Daniel S. Zachary

**Affiliations:** Johns Hopkins University, Krieger School of Arts and Sciences (Advanced Academic Programs), Washington DC, 20036 USA

## Abstract

A series of Poisson distributions are fit to sets of global cost-of-impact data representing large-scale accidents and anthropogenic catastrophes. The fits are used to build a function representing data means and are designated the Inverse Poisson Functional. Climate and environmental data have been used to develop a cost-frequency population distribution and to estimate the expected time between events. On a global scale, we show that expected wait- or reaction- times can be estimated using the Poisson density function. The functional is generated, representing the locus of means (peaks) from the individual Poisson distributions from different impact costs. Past (*ex-post*) forecasts relate to a range of natural and anthropogenic disasters; future (*ex-ante*) forecast presents global CO_2_ emissions. This paper shows that a substantial reaction to global climate change (CO_2_ emissions extremum) will occur in 55 to 120 years (95% CI) with a model prediction of 80 years.

## Introduction

According to the International Panel on Climate Change^1^ and the U.S. Department of Energy^2^, CO_2_ emissions are expected to rise at least through 2030. Although mitigation measures have been taken by several nations as communicated in the Paris Climate Summit^3^, no measurable lowering of post-Industrial Revolution CO_2_ concentrations has been observed to date. Furthermore, no direct solution on a global scale has been implemented or even agree upon that would change the current direction in the rise in anthropogenic CO_2_ emissions^4–6^. Average global temperatures are expected to rise within the range of 0.25 °C to 4.8 °C by 2100, with a likely increase of 1.5 °C according to the IPCC’s Fifth Assessment Report^1^. Other studies show that beyond 2.5 °C, the damage cost is projected to increase rapidly; indicative figures show a range between 7% to 25% of the global GDP for a warming of 5 °C^7^.

The numerous impacts of climate change (CC) include sea level rise, subsequent large-scale migration, drought, and coastal adaptation^8^. Studies project global sea level to rise by one to four feet by 2100, with an uncertainty range of 20 cm to 2 meters^9^. The’reaction path’ regarding how we deal with the impacts of CC has been determined using analytical methods, including integrated assessment models (IAMs).

The impacts from CC are linked not only to enormous costs related to the destruction and the adaption of infrastructure but also to health-related issues. Large-scale pollution has often been related to health impact. Some examples include sulfur-induced acid rain^10^, lead pollution resulting from the combustion of petroleum fuel containing lead^11^, and the stratospheric ozone hole resulting from chlorofluorocarbons (CFCs)^12^. These problems have been addressed in both regional and international mitigation agreements.

Regarding CO_2_ emissions and their impact, IAMs have long been used to perform simulations of the climate using reduced-order approximations to various Earth-systems. These models also include simulations of both the social science side (e.g., demographics, political, and economic modeling that affect greenhouse gas emissions) and the physical side (e.g., energy transfer, atmospheric, land, and ocean models that determine how and to what degree temperatures rise). IAMs have been used to explore economic scenarios and future CO_2_ levels and are considered valuable policy support tools^13–16^.

But despite the significant effort over the past few decades in developing these tools, straightforward mitigation approaches based on what we learn from IAMs have found resistance. The climate benefits from mitigation are realized only years after the costs are incurred, and the uncertainty of the results of mitigation inhibit the effort. The following statement from Hof *et al*^7^. summarize this concept,

The benefits of mitigation take place decades after the costs are made, and they are much more uncertain than the costs–as this depends on the possible occurrence of catastrophic events.

According to several authors, the uncertainties related to future climate are sufficiently large so that the results from a formal quantitative cost-benefit study (based on IAMs) would be unreliable^17–19^.

To further challenge the problem, global mitigation costs and benefits are unevenly distributed among countries. Fifteen to twenty nations are responsible for roughly 75 percent of global emissions, and the two major players are China and the USA^20^. This inequity reveals itself in the decision process and lends itself to non-cooperation in emissions reduction.

Given these challenges, this paper aims to focus on the progression of CO_2_ emissions in light of the historical evidence. We propose a descriptive approach to understanding the global behavior of CO_2_ emissions based on the cost-of-impact and the time required for the actual curbing of emissions as a result of measuring the mean response time of environmental impacts of similar costs. In other words, we propose that the delay of *substantial* mitigation to anthropogenic pollution is related to the time-delay of damage; the mitigation delay connects to the level of cost-impact from natural or anthropogenic disasters. We define substantial in this work by mitigation that curbs emissions so that concentration no longer increases, but begins to decrease (or at least levels to zero increase). Observation of environmental events, both natural and anthropogenic leads to the following two equivalence propositions:

1. Proposition 1 *The distributions of natural environmental events and anthropogenic accidents emanate from the same parent population and are defined by Poisson statistics*.
2. Proposition 2 *Emissions are the equivalent of a set of anthropogenic events; when the peak of emissions (highest impact cost) can be identified, it can also be analyzed statistically as an event*.

The paper will show the similarities in the underlying distributions of natural and anthropogenic events and then will use this information to build a time-delay distribution. We develop an extrapolation of time-delay and show examples of forecasts from both past (*ex-post*) and future (*ex-ante*). The paper is arranged in the following way: Section 1 proposes a look at global disaster data, both natural and anthropogenic; Subsection 1.1 illustrates fit functions for raw-count data and the time-delay data; Subsection 1.2 presents a model of time-delay; Subsection 1.3 presents *ex-ante* and *ex-post* forecasts. Finally, we present results (Section 2) and discuss interpretations (Section 3).

## Methods

### Data and fit functions

Data used to build the cost-frequency distribution is obtained from the International Disaster Database and is maintained at the Center for Research on the Epidemiology of Disasters (CRED^21^).

**Figure 1:**
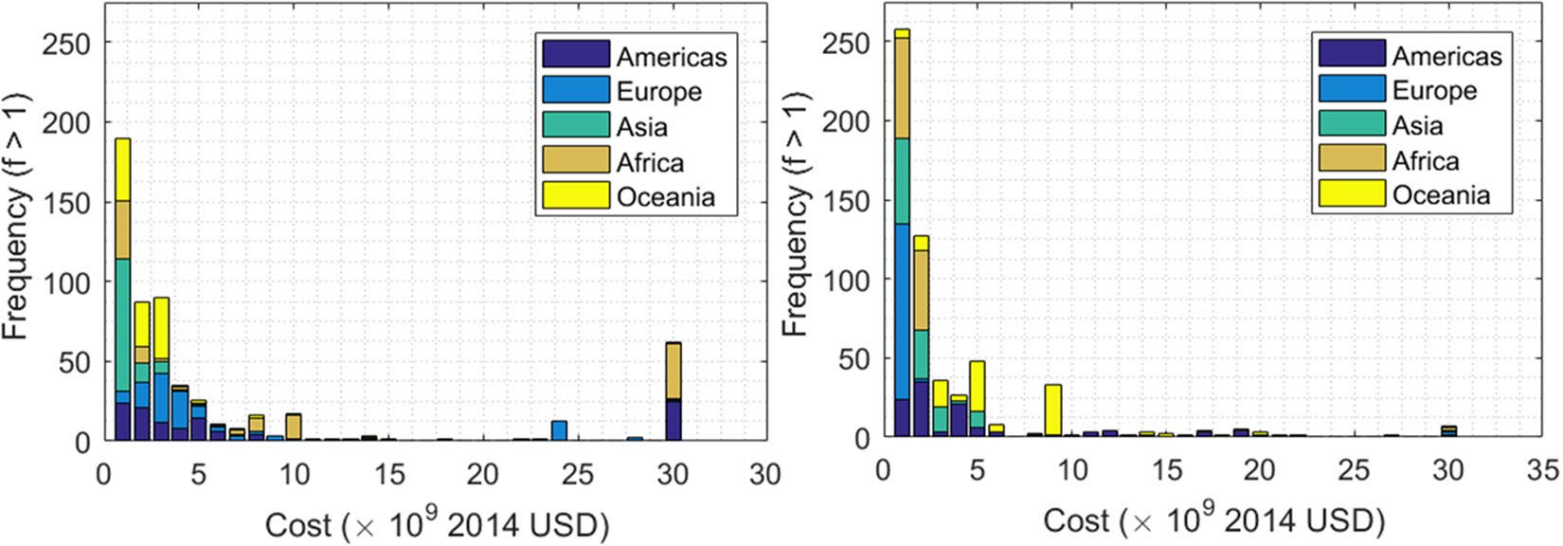
Overview of the environmental events (left) and anthropogenic accidents (right) displayed by continent.

The EM-DAT Database, containing 21,000 events is divided into natural catastrophes containing disaster subgroups of geophysical, meteorological, hydrological, climatological, and extra-terrestrial as well as technical accidents containing disaster subgroups of industrial, transportation, and miscellaneous accidents. Data are organized by cost (adjusted to the 2014 US dollar) and year. The raw data have been extracted and plotted in a frequency-stacked histogram shown in Fig. 1.

Raw count data of events for both natural and technical are tabulated from 1900–2016; These are designated as Nat-NE (natural events) and for the technical, Ant-NE (anthropogenic events). Cost data (adjusted to the 2014 US dollar) available from 1960–2016 are labeled as Nat-CE (natural, cost per event) and Ant-CE (anthropogenic, cost per event). Both natural and anthropogenic data show a monotonically decreasing trend with cost. Data sets are organized in Table 1.

**Table 1:** EM-DAT event organization.

Events	Type	Year	Acronym
Natural	Cost	1960–2016	Nat-NE
	Counts	1900–2016	Nat-CE
Anthropogenic	Cost	1960–2016	Ant-NE
	Counts	1900–2016	Ant-CE

**Figure 2:**
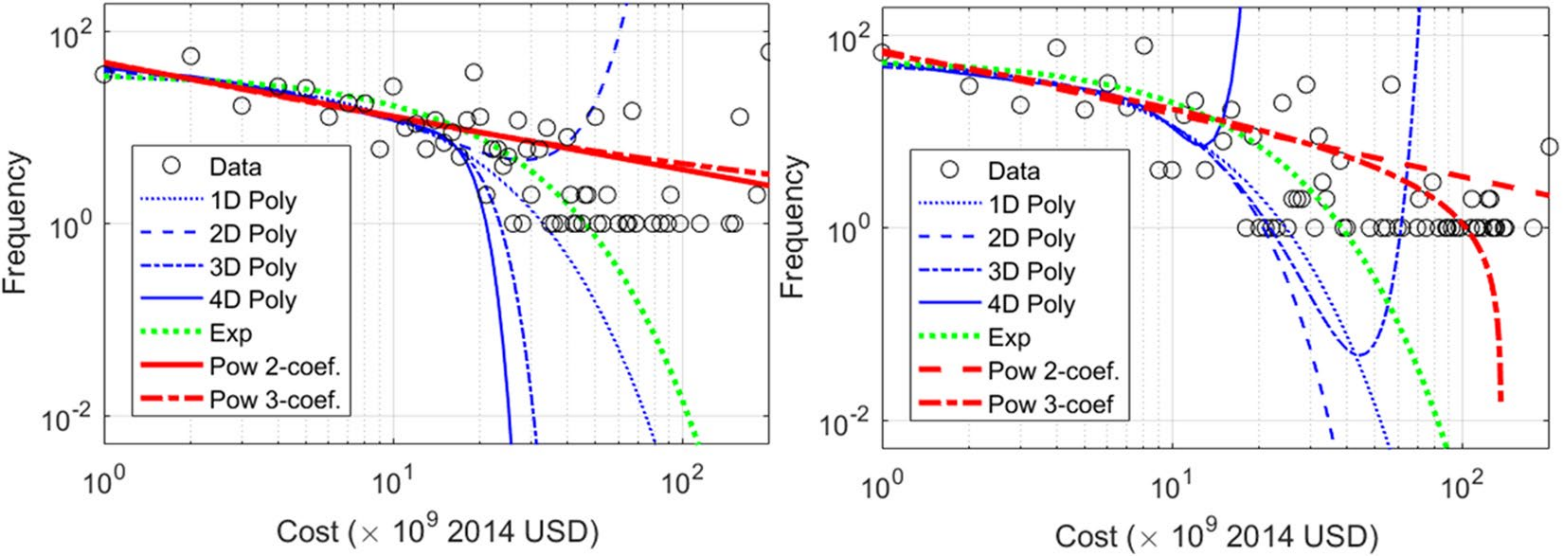
Frequency verses cost and several fit functions for natural (left) and anthropogenic (right) events.

**Figure 3:**
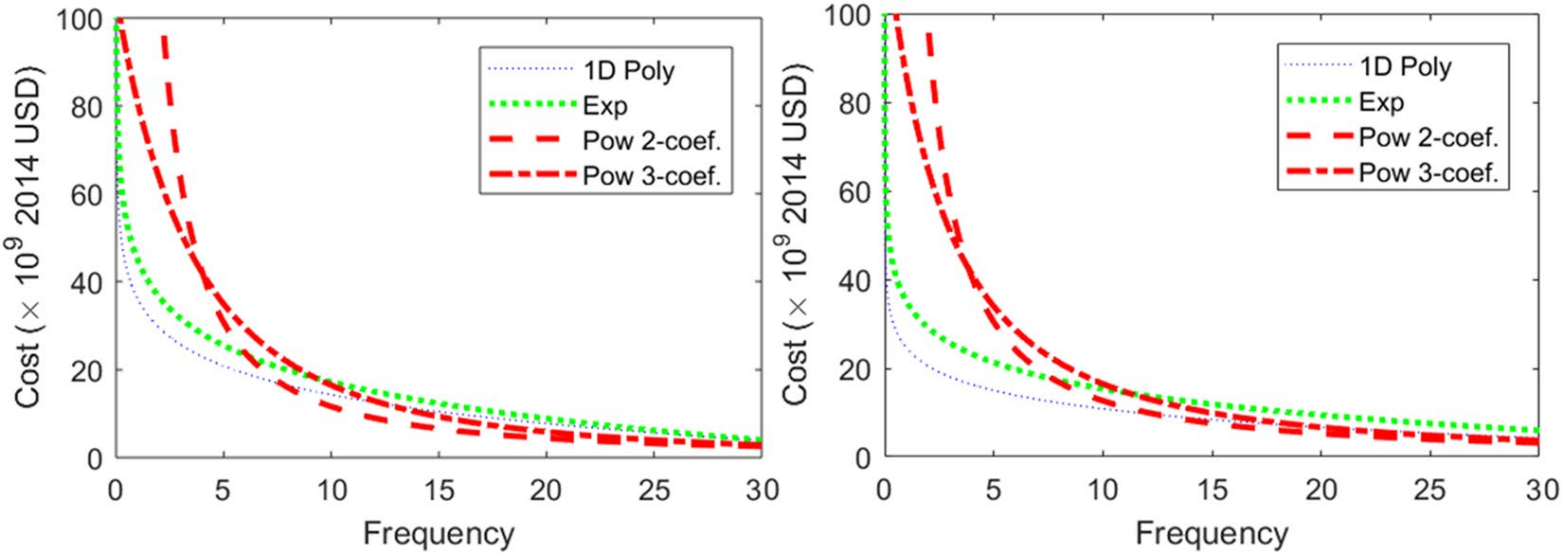
Cost frequency plots and fits for natural (left) and anthropogenic (right) data.

Frequency distributions of cost per event were developed to better understand the quality of the data and to help determine cut-off limits for the Poisson fits in the upcoming section. Fits were made with several functions including 1-, 2-, 3- and 4-dimensional (1D–4D) polynomials, *p*_1_*x*^*n*^ + *n* + *p*_2_*x*^*n*−1^ + … + *p*_*n*_*x* + *p*_*n* + 1_, *n* = 1,… 4, a 3-parameter exponential function with offset, *αe*^*η*^ + *γ*, and two power functions, *αx*^*β*^ and *αx*^*β*^ + *γ*′′. The data are displayed in a semi-log graph in Fig. 2 along the fits (only 3D and 4D polynomial fits are shown for clarity). The inverted plots are shown in Fig. 3. We use a Pearson’s *χ*-squared test,

1 $${\chi }^{2}=\sum _{i\mathrm{=1}}^{n}\frac{{({O}_{i}-{E}_{i})}^{2}}{{E}_{i}},$$

where *O*_*i*_ and *E*_*i*_ are the observable and expected frequency for data-point, *i* to determine the best fit for the data. The 1D–4D polynomial fits did the poorest and the inverse and power functions did the best to describe the data. The natural and anthropogenic data sets gave qualitatively similar results as shown in Table 2.

**Table 2:** Person’s *χ*-squared tests for exponential and power law fits.

Type of fit	Exp	PL 2-coef.	PL 3-coef.
Natural	1.2 × 10^6^	736	179
Anthropogenic	2.2 × 10^6^	1.16 × 10^3^	832

### The Delay Function

We define a probability distribution *P* such that, *P*({*k* events in the time interval *t*}) indicates the probability that *k* events are observed in a time interval of length *t*. Then,

2 $$P(k,t)=\frac{{(\lambda t)}^{k}{e}^{-\lambda t}}{k!}\mathrm{,\ }k=\mathrm{0,\ 1,\ 2,}\ldots \mathrm{.}$$

We assume that events are independent, non-simultaneous and have constant mean rates, characterizing the Poisson distribution^22^ with event rate *λ* with the property that the probability of an event in a small interval is proportional to the interval length.

**Figure 4:**
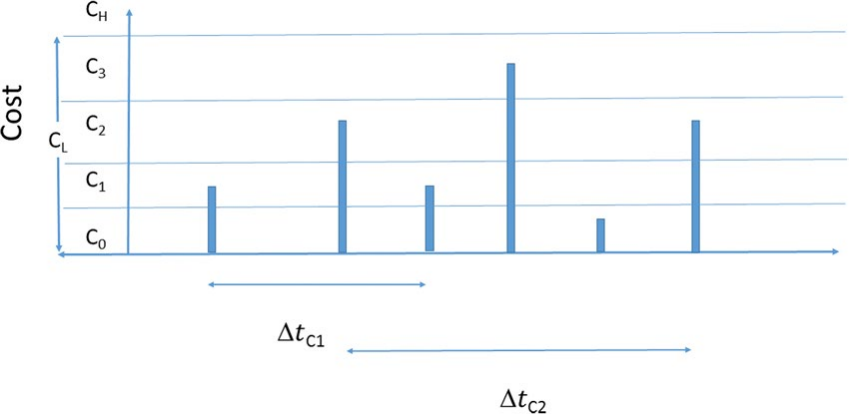
Sketch of a set of hypothetical events and Δ*t* of similar cost events.

To better qualify the data in Fig. 1, cost-cuts were produced by considering the level of impact for each event. The time-delay of similar events was determined by measuring the time (in years) between events in the same period (slices) as shown in the sketch of Fig. 4.

**Figure 5:**
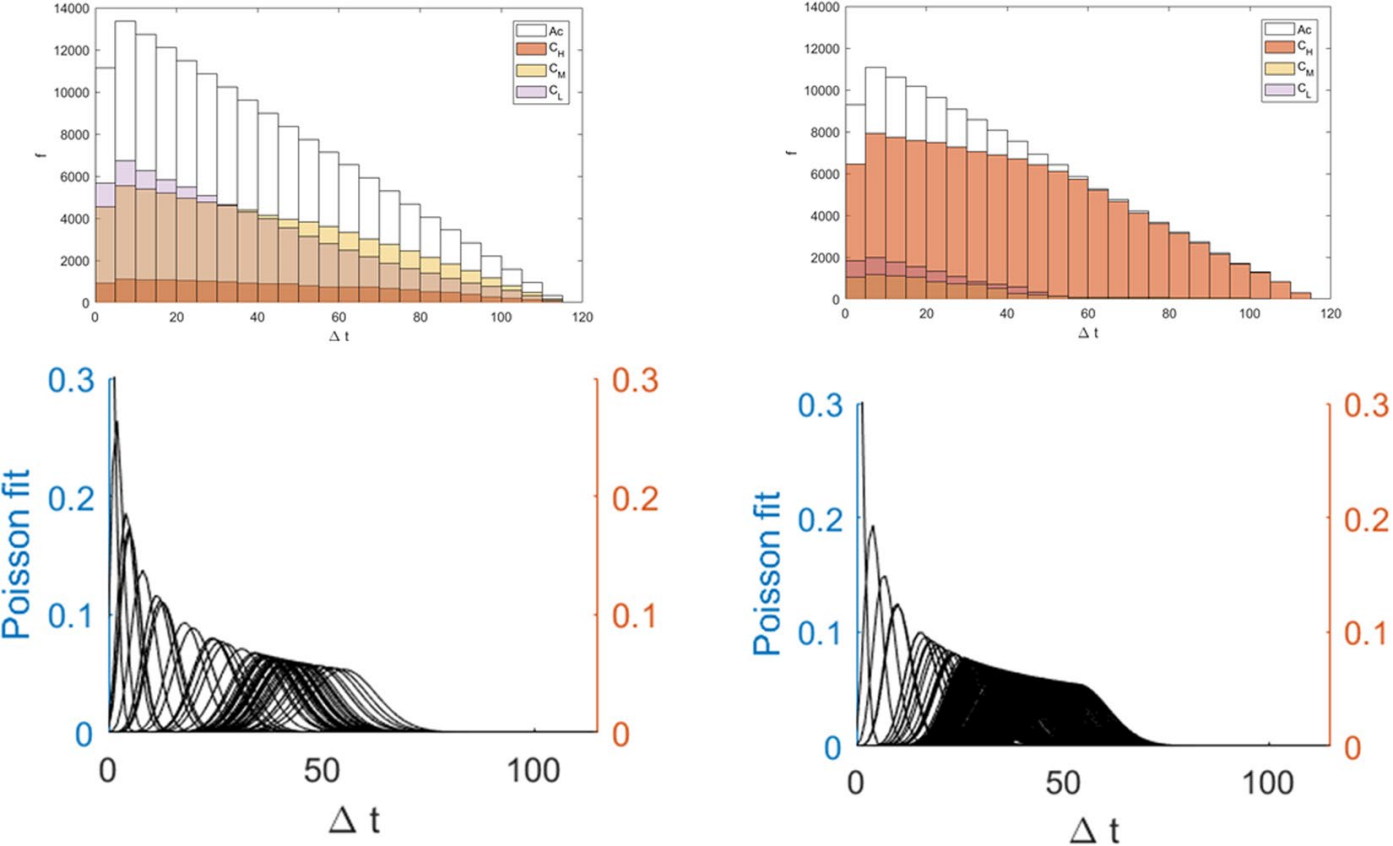
Frequency verses Δ t plots (top) with Poisson fits (bottom) for natural (left) and anthropogenic events (right).

Cost frequency data are partitioned into slices, *C*_*i*+1_−*C*_*i*_ = *C*_*S*_, where *C*_*S*_ is one billion (adjusted to 2014 USD). Three aggregate slices were also created, *C*_*L*_ < 4 × 10^9^ *USD*, $4\le {C}_{M} < 11\times {10}^{9}\,USD$, and ${C}_{H} > 11\times {10}^{9}$ USD (2014 adjusted). The distributions of delays for these aggregate slices are given along with the accumulative data in Fig. 5. Despite the overall shapes, anthropogenic distributions have much larger statistics in the *C*_*H*_ slice than do the natural events. The aggregated cuts, *C*_*L*_, *C*_*M*_ and *C*_*H*_, resulted in mean delays that increase with increasing cost slice *i*.

### The Inverse Poisson Functional

Now let *z* be the set of maximum probabilities *z* = P^*^(*k*, *t*) for the observable means, *λ*_*i*_, *i* = 1, 2, 3, …, the observed means from the Poisson fits for each slice *i*. This is a subset of a theoretical set of maximum probabilities $\widehat{z}=\widehat{P}\ast (k,t)$, where $z\subseteq \widehat{z}\in {\lambda }_{i}$ and where ${\lambda }_{i+1} > {\lambda }_{i},\forall \,i$. The function $\widehat{z}$ is monotonically decreasing with increasing cost slice *i*. The inverse function *z*^−1^ is convenient to plot as we are considering very large costs; we refer to this as the Inverse Poisson Functional (IPF).

## Results

### *Ex-ante* and *ex-post* fits

**Figure 6:**
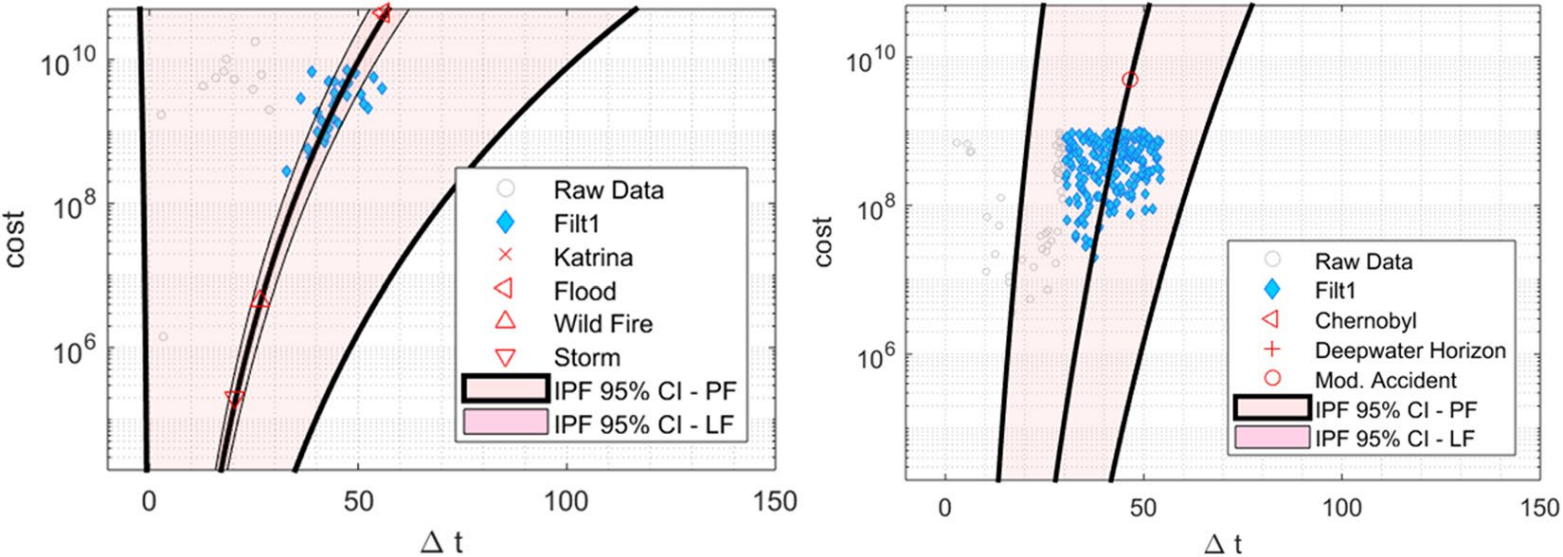
Delay plots for natural (left) and anthropogenic (right) accidents.

**Figure 7:**
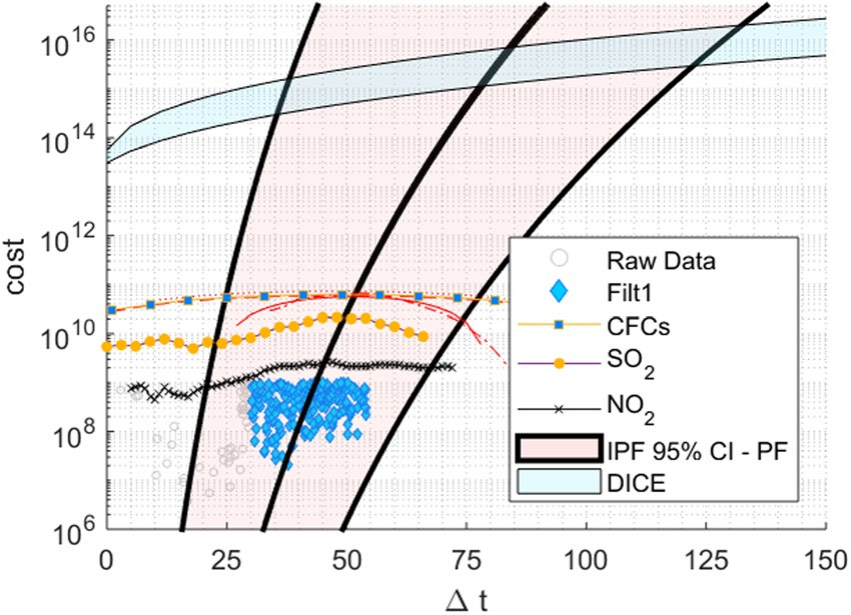
A climate delay plot for anthropogenic pollution events including both raw and filtered data points for CFCs, sulfur and nitrous oxide data and CC projections. DICE and CC model prediction (IPF 95% CI) are included.

The IPF is plotted for both natural and anthropogenic data in Fig. 6. Some specific natural events and man-made accidents are indicated in each figure. A series of statistical tests were conducted to produce an optimal set of fits. First, cost-cuts that gave insufficient statistics were filtered. Systematic tests were conducted to find a best filter cut for all three data-sets, natural, anthropogenic and combined. Poisson fits were improved when cuts had at least 100 counts and low-cost values inferior to 30 were filtered. Low statistics data (counts inferior to 100) accounted for 22% of the Natural data. By applying the cut, the standard deviation (SD) *s*, improved from *s* = 18.4 (raw data) to *s* = 5.38 (filtered). Similarly, 17% of the anthropogenic data had low statistics and when applying these two cuts, (SD) improved from *s* = 10.0 (raw data) to *s* = 6.22 (filtered), and combined data improved from *s* = 14.6 (raw data) to *s* = 6.23 (filtered). Increasing the count filter above 100 did not significantly improve the fits for any of the three data sets. For comparison, unfiltered data are shown in Figs 6 and 7.

Several events were chosen to test the model in *ex-post* fits. Comparisons made with large-scale natural events including Hurricane Katrina, 2005 (USA); the Great Thailand Flood of 2012 (Thailand); a San Diego Wild Fire of 1998 (USA); and a moderate storm with damage of 0.5 million (2014 USD levels). Anthropogenic accidents include the Chernobyl Nuclear event, 1986 (USSR, present-day Ukraine), the Deepwater Horizon Accident (DWH), in 2010 (USA) in the Gulf of Mexico (GOM), and a moderate industrial accident with damages of 0.5 billion USD (2014 adjusted). Costs for events were obtained from the International Disaster Database (EM-DAT). The delay time Δ*t*_*m*_ is obtained from the model and is shown for both types of events and their respective costs. Table 3 shows cost results and Table 4 shows results for delay times as well as the confidence intervals (CI). Climate delays are measured from 55–120 years using the combined data and from 50–165 years using only anthropogenic data.

**Table 3:** Accidents using cost data from EM-DAT and cost (2014 adjusted) impacts.

Type	Cost (×10^6^)	Δ *t*_*m*_ (years)	Symbol for Fig. 6	Description
**Natural**				
Hurricane	45	67	×	Katrina, Louisiana, USA, 2005
Flood	45.7	67	◁	Major Flood, Thailand, 2011
Fire	4.5	28	Δ	Wild fire, San Diego, CA, USA, 1998
Storm	0.5	26	▷	Moderate Storm damage
**Anthropogenic**				
Reactor meltdown	301	54	◁	Chernobyl, Ukraine, 1986
Oil Spill	60	52	+	DWH accident, GOM, USA, 2010
Industrial Accident	0.5	46	○	Moderate Industrial Accident

**Table 4:** Delay table with pollution and climate change impact levels (USA dollar, 2014 adjusted).

Emission	Δ *t*_*m*_	95% CI range (years)	*ϕ* Test	Symbol for Fig. 6	Description
CFCs	47	17–73	<1%	$-\square -$	Damage resulting from CFCs, 1920–2060
SO_2_	50	20–70	$\approx \mathrm{20 \% }$	$-\circ -$	Global SO_2_ damage, 1850–1999
NO_2_	47	25–65	≈20%	$-\times -$	Global NO_2_ emissions damage, 1970–2009
CC	80	55–120	<1%	$-\ast -$	Damage resulting from global CC

Pollution events are, of course, anthropogenic in nature, but additional consideration must be given since the pollutant occurs over some span of time. The time since the maximum emission of a waning pollution episode is analogous to the delay time. Likewise, the integrated value of the pollution event is analogous to the cost of a single event.

Several large-scale and pollution events were also examined. Damage from the diminution of stratospheric ozone and the subsequent creation of the Antarctic Ozone Hole has been measured prior to and following the establishment of the Montreal Protocol, representing an international cooperation in the reduction of CFC emissions with its ratification on 1 January, 1989^23^. Stratospheric ozone damage has been estimated in a study from Environment Canada presented to a UN meeting in 1997, “estimat[ing] a total CFC phase-out cost of $235 billion through the year 2060, but economic benefits totaling $459 billion.”^24^.

The main components of CFCs include: CFC-11 (F11), CFC-12 (F12), HCFC-22, and CFC-113 (dash-dash, solid line, dash-dot, dots respectively) and were all analyzed both separately and collectively. Pollutant projections have been made through 2100 and show a rise from approximately 1940–1990 and then a subsequent decline following 1990 to the end of the 21st Century^25^.

Sulfur-dioxide (SO_2_), resulting mostly from coal burning, has been present in significant quantities since the mid-1850s. This pollutant increased in the last part of the 19th Century and early 20th Century, peaked at about 75 million tons in the early 1980s and then declined^26,27^.

Nitrous-oxide emission has also occurred over approximately the same time span as sulfur emissions, but reliable data are plotted only since the early 20th Century in Fig. 7. Again a steady rise in emissions has occurred, but unlike sulfur, nitrous oxide emissions continue to rise.

The impact of both sulfur and nitrous oxide emissions has been evaluated using a cost-per-ton estimate of 10,860 USD and 1780 USD for sulfur and nitrous oxides emissions respectively^28^. These costs are adjusted for inflation and calibrated to the 2014 USD value^29^.

The impact cost per year (2014 adjusted) is plotted in Fig. 7. The pollution values were superimposed on the IPF curve. The depreciating value of the dollar resulted in a diminishing value in impact; NO_2_ emissions, which are still rising, show a low peak when multiplied by this factor.

Finally, a quality test was developed to compare the pollution events to the statistical test. A factor *ϕ* is defined,

3 $$\varphi ={f}_{E}/{T}_{E},$$

where *f*_*E*_ is the fraction of emissions before the 0-point on the Δ*t* scale (x-axis in Fig. 7) and *T*_*E*_ is the total emissions throughout the history of the pollutant (the 1850s in the case of SO_2_ and NO_2_) to the present. This factor indicates the accuracy of the IPF in matching the peak curve.

Chlorofluorocarbons, for example, show a sharper rise in output per year and peak much faster than the industrial pollutants *SO*_2_ and *NO*_2_. The commercialization of large-scale chlorofluorocarbons originated in the 1930s by the DuPont Corporation, using the commonly known brand-name Freon. As a result, CFCs having a sharp rise in the 1940s and peaking in the late 20th Century give an accurate match (*ϕ* < 1%) to the IPF. The ratification of the Montreal Protocol (1989) took place approximately 55 years after the commercialization, similar to the *ex-post* prediction of 50 years. On the other hand, both *SO*_2_ and *NO*_2_ projections give *ϕ* ≈ 20%, indicative of the extended period (beginning in the mid-1850s to the present) of small emissions in the early phase of the industrial revolution.

Only after considering a cascade of consequences can the cost of CC impact be determined. IAMs have been developed to make these calculations, and generally, the critical issues are the projected levels of carbon dioxide and other GHG emissions such as methane, nitrous oxides, as well as CFCs. The latter three gases are much more potent regarding global warming potential but are also significantly less present in the atmosphere^30^. Estimations have been made using energy model projections, macroeconomic modeling and estimates of future GDPs in the Dynamic Integrated Climate-Economy model, DICE, by Nordhaus, Weitzman and associates^31–34^. This particular model is considered a prominent tool used for global CO_2_ calculation and integrated assessment and is used by the United States Environmental Protection Agency^35^. Nordhaus *et al*. present a schematic of avoided damages and costs through mitigation schemes, and this is compared to a scenario without mitigation. Global temperatures are expected to increase by 3.5 °C by 2100 and 5.4 °C by 2200 in different versions of this model. *Ex-post* predictions are made and a relatively small quality factor, *ϕ* < 1% was determined, indicative of the relatively small damage that has already occurred. The sharp rise in future damage is predicted using DICE^16^, and this may lead to similarities with the CFC scenarios. Predictions from the model show an 80-year time-delay and a 55–120 year in a 95% CI. See Fig. 7.

## Discussion

Climate change can be considered a continual set of events that theoretically leads to a single concentration peak of CO_2_. By using the assumption that pollution events are intrinsically a series of anthropogenic events, starting from small emissions and leading to an eventual peak, we were able to use historical data and extrapolate to make a forecast. This was achieved by using *ex-post* comparisons with large-scale events (CFCs, SO_2_ and NO_2_) and then to making the natural extension to an *ex-ante* prediction for CO_2_ emissions. Given sufficient statistics at a cost level, Poisson distributions could be generated and extrapolated to high costs, namely the high cost of CC. Also, the study showed that within a 95% CI, the time delay of natural events and anthropogenic accidents is similar and therefore statistics could be combined from both data sets, improving the accuracy of the extrapolation.

Many assumptions are required to forecast environmental and climate events besides statistical limitations, not the least of which are the assumptions in the CC modeling, both from the physical perspective (e.g., uncertainties in atmospheric and energy process) and from the social-economic perspective (e.g., discounting rates).

This study combines the forecasts from the statistical model with the forecast made with DICE, which views the economics of CC in a neoclassical economic growth theory^36^ and extends the concept of capital by treating emissions as’negative capital’. The DICE model also allows for scenarios of emissions reductions: “economies [can] reduce consumption today [to] prevent economically harmful climate change and thereby increase consumption possibilities in the future”^37^. But the uncertainties of the cost of damage as a result of carbon are difficult to address though they have been discussed in several studies involving DICE and other IAMs^38,39^.

When considering pollutant delay times, chemical lifetimes of GHGs also needed to be considered. Two metrics are often used to measure atmospheric gas lifetimes. Global mean lifetimes (GML) characterize the time required to turn over the global atmospheric burden (teragrams of pollution divided by the mean global sink, teragrams/yr, for a gas in steady state)^6^. A second metric is the perturbation lifetime (PLT), a measure of the time where anomalous levels of, CO_2_, for example, remain in the atmosphere; PLT is also the time required for a fractional reduction to 1/*e*^40^. For completeness, both GML and PTL values are considered whenever possible. Chlorofluorocarbon GMLs range from 10 to 100 years: CFC-11 (F11) 45 years, CFC-12 (F12) 100 years, HCFC-22 11.9 years, CFC-113 85 years and nitrous oxides 120 years (GML) years or 114 years (PLT)^41^. No single lifetime can be defined for CO_2_ as there are several processes that remove carbon dioxide from the atmosphere. “Between 65% and 80% of CO_2_ released into the air dissolves into the ocean over a period of 20–200 years. The rest is removed by slower processes that take up to several hundreds of thousands of years^42^. Only sulfur dioxides lifetimes are short; lifetimes of SO_2_ in the troposphere are a few days^43^. Extended gas lifetimes cause damage impact to be extended. This fact is taken into account by measuring the concentrations of species. Longer lifetimes result in a slower decrease of the pollutant concentration as shown in Fig. 7. Similarly, even if CO_2_ emissions were stopped completely and instantaneously, the delay time would cause a fairly slow decrease for pre-industrial levels. According to a study funded by the Swiss National Science Foundation, “Within a millennium of this simulated shutoff, the carbon itself faded steadily with 40 percent absorbed by Earth’s oceans and land masses within 20 years and 80 percent soaked up by the end of the 1,000 years”^44^.

Another limitation is the open question of how to assign damage. The DICE models, for example “[assign] monetary values to the benefits of climate mitigation on the basis of incomplete information and sometimes makes speculative judgments concerning the monetary worth of human lives and ecosystems while downplaying scientific uncertainty about the extent of expected damages”^45^. To be fair, this issue is also present in other IAMs as well and shows that how we monetize damages, either present or future, will shape strategies.

Uncertainties are also compounded the further out climate predictions are made. According to the US Global Change Research Program^8^ and the IPCC, 5th Assessment Report^1^, uncertainties in CC are related to uncertainties in (1) observations; (2) response of the drivers of CC; (3) understanding the recent changes in the climate system due to anthropogenic influences; and (4) the projections of global and regional CC due to the incomplete understanding of the total climate system.

The statistical model presented here avoids some of the problems associated with IAMs as the model only requires a single cost assumption for its prediction and is insensitive to the details of cost calculation. The quality factor *ϕ* allowed for testing of past pollution events can allow some degree of confidence in the predictive power of the model based on the evolution of impact, namely the rate at which it grows. It appears, at least qualitatively, that CC resembles the impact evolution of CFCs as evidenced by the close match between the model and the observed time from onset to ratification of the Montreal Protocol. The model performed less well in *ex-post* comparisons with SO_2_ or NO_2_ emission scenarios ($\varphi \,\approx$ 20%).

The study also looked at the global situation. By viewing the ’big picture’, we remove the small-scale issues that make global projections more challenging (e.g., except for the largest polluters,  individual nation’s policy decisions to either favor or not favor low carbon emissions are individually too small to be considered in this model). However, we do not imply that individual nation’s efforts are not important. According to the IPCC’s Fifth Assessment Report, “Effective mitigation will not be achieved if individual agents advance their own interests independently”^46^. The results of this study are therefore consistent with the IPCC’s assessment that cooperative effort is mandatory. “Collective and significant global action is needed to reduce greenhouse gas emissions to keep global warming below 2 °C. The report also states “that the longer we wait, the more expensive and technologically challenging meeting this goal will be”^46^. The study is also qualitatively consistent with carbon emission decreasing by using an optimal carbon tax scenarios for standard and comprehensive DICE model simulations. These simulations show 100 and 50 years for carbon emission subsiding, respectively^39^.

The importance of global cooperation to achieve carbon emissions reduction is underlined by a recent Price Water Cooper report^47^. The failure of even-handedness in sharing the cost of reducing carbon is evident. Some countries are dealing with the problem. Australia, for example, has reduced carbon emissions by 7.2% between 2012 and 2013. Certain countries such as the U.K., Italy, and even China have all managed impressive decarbonization rates between four and five percent. Other countries, including the U.S., have increased their carbon emissions. The study indicates the trend “[f]or the sixth year running, the global economy has missed the decarbonization target needed to limit global warming to 2 °C. Confronted with the challenge in 2013 of decarbonizing at 6% a year, we managed only 1.2%“^47^. A’climate’ of non-cooperation seems to be the route the US is taking since 2017 as is evidenced by it’s withdrawal from the Paris Climate Agreement^48^. Consistent with this study, we highlight the concept that when pollution does not directly affect the individual (or polluter) at least directly (spatially detached) or right now (temporarily detached), the results are, on a global scale, inaction. This reality is analogous to the concept “Not In My Back Yard” (NIMBY) or in this context, *My Neighbor’Oughta’ Pay* (MNOP). This action, or lack thereof, perhaps cynical, can be viewed as a self-interest scenario and is rational, at least in the short-term. In this scenario, the pollution problem is ignored until the cost of impact is sufficiently large, that action must then be taken. This reasoning is consistent with the cost analysis of other anthropogenic pollution issues in the past.

On the other hand, waiting for the impact of CC to increase before responding may have additional consequences that were not present in previous pollution scenarios. Compounding damages are of concern for CC; the longer we wait to do something the more expensive will be the cost of impact and the cost of mitigation. The increase will not be linear as a function of CO_2_ emission but will rise in a compounding fashion. According to an assessment by the Netherlands Environmental Assessment Agency, “Mitigation costs will exceed the avoided damage in the short term, but eventually, the benefits of reduced climate change will outweigh the mitigation costs.” From the same report, “In the second half of this century, without mitigation efforts, adaptation costs are likely to increase sharply, and large residual damage will remain unsolved, as adaptation can only reduce them to a certain degree”^16^.

## Conclusion

The Inverse Poisson Functional has been compared in *ex-ante and ex-post fits*. The model is in direct agreement with the historical time-delay data for CFC emissions onset and the ratification of the Montreal Protocol. The model therefore gives reasonable fits to global pollution events for chlorofluorocarbons and to a lesser degree for sulfur and nitrous oxides emissions. The model also predicts a decrease in CO_2_ levels in 80 years and a 95% CI spread of 55−120 years. Observations of emissions scenarios from past events can be used in a statistical model to extrapolate and therefore predict future peaking of CO_2_ emissions. The statistical model presented here allows for a method relatively independent from IAMs to determine the global reaction time to climate change and presents a view based on observation. Some scholars take a more normative approach to CC, assigning a damage to emissions in IAM simulations and therefore curbing emissions faster^49–52^.
